# JRR Award at ICRR 2015

**DOI:** 10.1093/jrr/rrv060

**Published:** 2015-09-21

Eleven prizes were awarded to outstanding oral presentations given at ICRR 2015, which covered topics relevant to JRR. The winners were announced at the ICRR 2015 closing ceremony, and each received a certificate and small gift, as well as the offer of waived publication charges for their next submission to JRR. The eleven winners from 2015 are listed below.


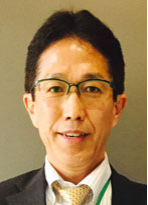


**Prof. Hiroshi Onishi** (University of Yamanashi, Japan), Japanese multi-institutional study of SBRT for more than 2000 patients with stage I non-small cell lung cancer or pulmonary oligometastases.


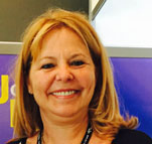


**Prof. Claire Rodriguez Lafrasse** (EMR3738, France), Cancer Stem cells and EMT: Guilty of HNSCC recurrences but condemned by the combination of carbon ion irradiation and EGFR-inhibition.


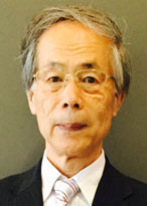


**Mr. Haruo Tsuruta** (The University of Tokyo, Japan), Retrieval of atmospheric radiocesium after the Fukushima accident by analyzing filter-tapes of operational air quality monitoring sites.


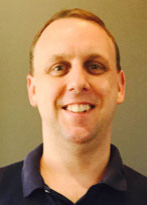


**Dr. Anthony J Davis** (University of Texas Southwestern Medical Center, United States), BRCA1 modulates DNA-PKcs autophosphorylation to suppress NHEJ in S phase of the cell cycle.


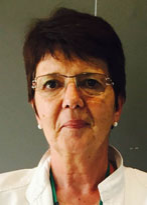


**Dr. Sylvia Ritter** (GSI Helmholtzcentre for Heavy Ion Research, Germany), Effects of X-rays and C-ions on early embryonic development.


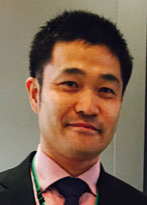


**Dr. Kentaro Fujii** (Japan Atomic Energy Agency, Japan), Physical process of decomposition of hydrated deoxyribose by oxygen K-shell ionization.


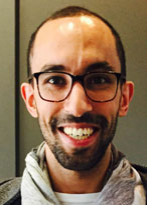


**Mr. Hakim Belmouaddine** (University of Sherbrooke, Canada), Low energy secondary electron-induced DNA damage through femtosecond laser pulse filamentation in aqueous solutions.


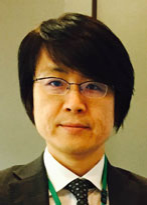


**Dr. Takashi Moritake** (University of Occupational and Environmental Health, Japan), RADIREC: System for mapping and collecting entrance skin dose during vascular interventional radiology.


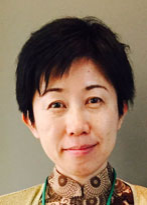


**Dr. Ritsu Sakata** (The Radiation Effects Research Foundation, Japan), Radiation risks of upper digestive cancers in the cohort of atomic-bomb survivors.


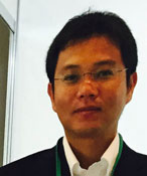


**Dr. Takuya Maeyama** (RIKEN, Japan), Radiological properties of the nanocomposite Fricke gel dosimeter for heavy ion beams.


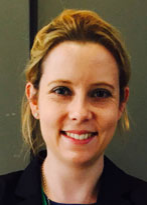


**Dr. Julia Hess** (Helmholtz Zentrum Muenchen, German Research Center for Environmental Health GmbH, Germany), CLIP2 as radiation biomarker in papillary thyroid carcinoma.

